# Plant Fortification of the Diet for Anti-Ageing Effects: A Review

**DOI:** 10.3390/nu12103008

**Published:** 2020-09-30

**Authors:** Daljeet Singh Dhanjal, Sonali Bhardwaj, Ruchi Sharma, Kanchan Bhardwaj, Dinesh Kumar, Chirag Chopra, Eugenie Nepovimova, Reena Singh, Kamil Kuca

**Affiliations:** 1 School of Bioengineering and Biosciences, Lovely Professional University, Phagwara 144411, Punjab, India; daljeetdhanjal92@gmail.com (D.S.D.); sonali.bhardwaj1414@gmail.com (S.B.); chirag.18298@lpu.co.in (C.C.); 2 School of Bioengineering and Food Technology, Shoolini University of Biotechnology and Management Sciences, Solan 173229, Himachal Pradesh, India; mails4sharmaruchi@gmail.com (R.S.); dineshkumar@shooliniuniversity.com (D.K.); 3 School of Biological and Environmental Sciences, Shoolini University of Biotechnology and Management Sciences, Solan 173229, Himachal Pradesh, India; kanchankannu1992@gmail.com; 4 Department of Chemistry, Faculty of Science, University of Hradec Kralove, 50003 Hradec Kralove, Czech Republic; eugenie.nepovimova@uhk.cz

**Keywords:** anti-ageing, diet, eating habits, functional foods, skin ageing

## Abstract

Ageing is an enigmatic and progressive biological process which undermines the normal functions of living organisms with time. Ageing has been conspicuously linked to dietary habits, whereby dietary restrictions and antioxidants play a substantial role in slowing the ageing process. Oxygen is an essential molecule that sustains human life on earth and is involved in the synthesis of reactive oxygen species (ROS) that pose certain health complications. The ROS are believed to be a significant factor in the progression of ageing. A robust lifestyle and healthy food, containing dietary antioxidants, are essential for improving the overall livelihood and decelerating the ageing process. Dietary antioxidants such as adaptogens, anthocyanins, vitamins A/D/C/E and isoflavones slow the ageing phenomena by reducing ROS production in the cells, thereby improving the life span of living organisms. This review highlights the manifestations of ageing, theories associated with ageing and the importance of diet management in ageing. It also discusses the available functional foods as well as nutraceuticals with anti-ageing potential.

## 1. Introduction

**Figure 1:**
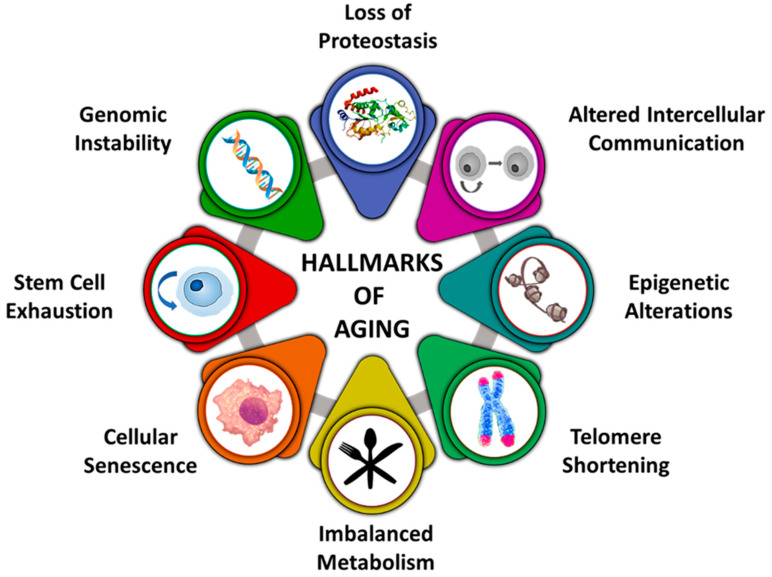
Hallmarks contributing to ageing.

Ageing is a progressive biological process which affects the normal functions of cells and tissue, thereby imperiling the person towards diseases and mortality [1]. For a layman, it is the process of maturing and growing old. Both internal and external factors play an integral role in ageing [2]. Internal factors comprise the usual biological processes of the cell, whereas the external factors involve chronic sun-exposure, hormonal imbalance, nutritional deficiencies, ultraviolet (UV) irradiation and other factors such as pollution and smoking [3]. The hallmarks associated with ageing have been illustrated in Figure 1. Skin ageing, characterized by wrinkling, can be reduced via suitable preventive measures involving the consumption of antioxidant-rich supplements, a balanced diet and undertaking skincare [4]. By opting for these measures, the harmful effects induced by free radicals can be restrained [5].

Over the past few decades, the relationship between nutrition and ageing has been extensively studied in both animals and humans [6]. Nutraceuticals are nutritional elements with medicinal characteristics; hence the name, where “Nutra” stands for food and “ceutical” means therapeutic properties [7]. As per the definition of Foundation for Innovation in Medicine (FIM), nutraceuticals are the “food and food products” that have medicinal value and provide health benefits, especially in preventing and treating age-related diseases [8]. These products include functional foods, dietary supplements and herbal extracts, which provide health benefits in the long-run when consumed as supplements in the diet [9]. Even researchers have suggested that antioxidants have propitious effects on both chronic as well as age-related diseases, especially neurodegenerative diseases and cancer [10]. Various food supplements that exhibit an antioxidant potential, such as carotenoids, flavonoids and vitamins, prevent and treat ROS-associated chronic conditions, which results in healthier and longer lifespans [10]. Food supplements produce antagonistic effects against the degenerative and inflammatory processes in the body, and have beneficial effects on the immune and digestive system, hence improving the quality of life [11].

The current review focusses on highlighting the manifestations of ageing and theories associated with ageing. Additionally, it also discusses the importance of diet management in ageing and functional food, as well as nutraceuticals with anti-ageing potential.

## 2. Manifestation of Ageing

Clinical manifestations of intrinsic ageing can be determined by assessing the regenerative ability of the damaged tissues or organs [12]. All dividing and differentiating cells are vulnerable to insults causing intrinsic ageing [13]. The visual traits of ageing start appearing in the early 40s. Most cells, tissues and organs steadily undergo ageing and become incompetent [14]. A significant effect can be observed on the skin, which turns loose, thin wrinkled and inelastic [15]. The face fat also reduces, leading to hollowed eye sockets and cheeks.

Furthermore, the hair starts thinning from the armpits, pubic area and scalp [16]. As melanin content decreases, the hair strands become thinner grey, and the nails become thinner as well [17]. At over 80 years old, more noticeable visual changes can be observed, such as the compression of spinal disks, vertebrae and joints. The hearing abilities also diminish depending on the severity of the ageing phenomenon [18].

Other than this, the elderly population gets presbyopia and may require reading glasses [19]. In comparison to healthy adults, they lack deep sleep and are unable to take sufficient rest as required by the body at this stage [20]. The bone density decreases and becomes weaker, increasing the risk of fracture [21]. Due to slow metabolism and hormonal changes, there is a reduction in muscle mass and an increase in body fat [22]. Besides this, older adults also suffer from lapses of memory and vagueness, preventing them from recalling names and memories [23]. The heart and lungs become less efficient with time, and kidney functions are abated [24]. The accumulated harmful metabolic waste later appears as dangerous diseases and allergies, causing significant discomfort to older people [25]. Moreover, females at menopause produce reduced amounts of estrogen, due to which they experience various changes, such as vaginal dryness, hot flashes, chills, night sweats, sleeping problems, mood swings, weight gain and slowed metabolism [26]. Besides this, an unhealthy diet and indolent lifestyle further increase the risk of occurrence of chronic diseases in elderly people, such as cancer, osteoarthritis, type 2 diabetes, obesity, coronary artery disease osteoporosis and high blood pressure [27].

## 3. Theories of Ageing

Several theories have been formulated to define the ageing phenomenon. These theories have been postulated based on certain assumptions, but none of them provide a satisfactory explanation [28]. There are three major theories for ageing, i.e., genetic theories, dysfunction of interlinked organs and physiological approaches [29]. Of these, three physiological theories have been extensively studied, which comprise the cross-linking theory, the waste material accumulation theory and the free radical theory [30].

In 1950, Denham Harman stated that ageing is the result of the massive production of free radicals [31]. In general, free radicals are those atoms or molecules that have unpaired electrons and possess the ability to form electronic couples [32]. This explains the short life and high reactiveness of these molecules. These free radicals are usually formed during the metabolic reactions under normal conditions [33]. Moreover, the generation of these free radicals also takes place during exposure to cigarette smoke, UV rays and toxic substances, as well as during emotional stress [34]. Even though free radicals are involved in normal metabolic processes, but they do not generally infiltrate the cells. Still, when they do, they have harmful and deleterious effects on various organs [35].

**Figure 2:**
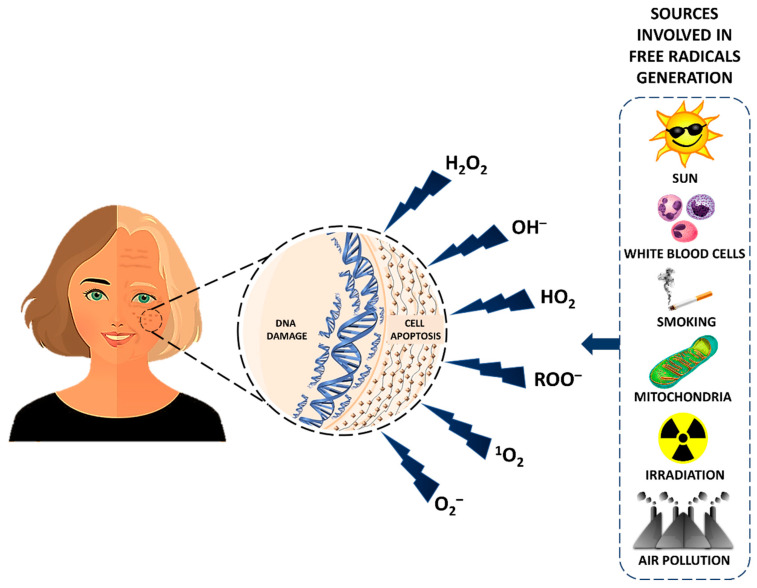
Schematic representation of sources involved in the formation of free radicals and their association with the ageing process.

Free radicals released from food are essential for energy production within the cell [36]. Additionally, their production also protects the body from opportunistic infections and elicits the synthesis of hormones involved in effective communication within the body [37]. However, the excessive production of free radicals has detrimental effects on DNA, collagen, elastin and blood vessels [38]. Oxidative damage to different biomolecules, such as DNA, macromolecules and proteins, takes place over time [39]. It is considered a significant factor, but is not the only factor responsible for ageing [40]. Fundamentally, oxygen has a dual role in our body, i.e., it is necessary for life and is one of the chief components of harmful compounds like free radicals [41]. Free radicals are generated by the aerobic metabolism. They liberate different types of reactive oxygen species, such as singlet oxygen (^1^[O_2_]), superoxide anion radicals (O_2_^–^), hydroxyl radicals (OH^–^), hydroperoxyl radicals (HO_2_), peroxide radicals (R = lipid) (ROO^–^) and hydrogen peroxide (H_2_O_2_) [42]. The various sources involved in the generation of free radicals are illustrated in Figure 2.

For example, if the free-radical-mediated DNA mutations are left uncorrected via repair mechanisms, this defect persists even after successive replication cycles, transcription and translation [43]. It is well-known that free radicals are formed by the aerobic metabolism for the synthesis of energy-rich molecules like ATP, which are synthesized in mitochondria (also known as cell factories) [44]. As humans start ageing, the efficacy of mitochondria in synthesizing ATP substantially decreases, thereby allowing the accumulation of free radicals in mitochondria as well as permitting the passage of free radicals through the mitochondrial membrane, thereby damaging other parts of the cell [45]. These alterations have helped to determine the key factors which favor ageing, i.e., increases in oxidative stress and a decrease in energy production [46]. Even the published literature has stated that a high degree of mutation is observed in mitochondrial DNA in contrast to nuclear DNA due to oxidative stress [47]. Therefore, calorie restriction (CR) impedes the process of ageing and increases the lifespans of flies, fish, spiders and mammals (mice and rats) [48]. This happens because CR decreases the oxidative load, which reduces the free radical formation in mitochondria [49]. The reduction in the free radical formation substantially reduces the number of oxidized proteins, lipids and mutated mitochondrial DNA [50]. Extensive studies have been conducted on rodent models to assess the effects of a diet enriched with minerals and vitamins in ageing [44]. As such, it is believed that calorie restriction and the consumption of food rich in antioxidants can considerably prolong the life span of individuals [51].

An important theory that explains the process of ageing is the shortening of the telomeres. Due to the end-replication problem, the telomeres are shortened in every generation of the cell till they reach a critical length in the crisis stage of ageing [52]. At this stage, the cell division slows down considerably, causing the cell to slowly die. This may be referred to as “replicative mortality”. Cells involved in growth, development and reproduction express high levels of the enzyme telomerase, which maintains the length of the telomeric DNA [53]. These cells include the stem cells and reproductive cells (eggs and sperms). However, most adult cells have low expressions or no expression of telomerase, which causes these cells to age and eventually die [54].

## 4. Plant-Based Supplements with Anti-Ageing Potential

Plants and their inherent components are well known to exhibit antioxidant potentials, sch as carotenoids, flavonoids and vitamins, that aid in the prevention and treatment of ROS-associated chronic conditions [55]. These supplements have antagonistic effects against the degenerative and inflammatory processes in the body and show beneficial effects on the immune and digestive system, hence improving the quality of life [56]. Some of the predominantly used plant-based supplements have been discussed below.

### 4.1. Adaptogens

Adaptogens are compounds obtained from herbal plants for maintaining homeostasis and stabilizing the physiological processes in humans [57]. These compounds reduce cellular sensitivity to stress and improve the ability of the body to resist the damage from other risk factors [58]. Moreover, they also help in restoring and promoting normal physiological function [59]. A few of the highly known adaptogens have been discussed below.

### 4.2. Bacopa monnieri

*Bacopa monnieri*, also known as Brahmi, is a perennial herb with small oblong leaves and purple flowers [60]. Highly valuable nootropic phytochemicals, such as bacosides, are found in this medicinal herb [61]. Brahmine and Herpestine are the two essential phytochemicals that are predominantly extracted from this herb [62]. The phytochemicals obtained from Brahmi aid in protecting the brain from the attack of free radicals and stimulating cognitive functioning and learning [63]. It has been comprehended that the regular consumption of Brahmi oil reduces the chance of various diseases like Alzheimer’s disease and amnesia [64]. Bhattacharya et al. (2000) found that extracts of *Bacopa monnieri* enhance the activity of reactive oxygen species-scavenging enzyme catalase (CAT), glutathione peroxidase (GPX) and superoxide dismutase (SOD), in a dose-dependent manner. This study was carried out in the brain regions of rats and investigated after 14 and 21 days [65]. Shinomol and colleagues conducted an in vitro and in vivo study using 3-nitropropionic acid (NPA) (fungal toxin responsible for causing neurotoxicity in humans and animals) and *Bacopa monnieri* extract. The result obtained showed that NPA was effective in inducing the oxidative stress in dopaminergic (N27) cells and mitochondria of the striatum of rats, whereas *Bacopa monnieri* extract was found to be effective in regulating the NPA-induced oxidative reactions and reducing the Glutathione (GSH) and thiol levels [66]. Kumar and his colleagues also conducted a six-week randomized placebo-controlled trial to assess the effect of *Bacopa monnieri* extract on the cognitive functions of students studying medicine. The result obtained from the study showed significant improvement in the cognitive functioning of the students [67].

### 4.3. Curcuma longa

*Curcuma longa* is a plant of the ginger family that produces a compound known as curcumin [68]. It is known for diverse biological activities, such as its anti-cancerous, anti-inflammatory and antioxidant properties [69]. Due to these natural properties, curcumin is a potential therapeutic agent for treating different types of cancers [70]. Many studies have revealed that curcumin can suppress the expression or activity of cyclooxygenase-2 (COX-2), prostaglandin E2 (PGE2), pro-inflammatory cytokines and tumor necrosis factor-α (TNF-α) [71]. The antioxidant properties of curcumin can aid in the reduction of ROS production, the scavenging of free oxygen radicals and obstructing lipid peroxidation [72]. The consumption of curcumin via the oral route in rodents has been shown to ameliorate cystic fibrosis and block tumor progression; still, the evaluation of humans is pending [70]. A study reported that curcumin induces a cellular stress response in human fibroblasts via redox signaling and the phosphatidylinositol 3-kinase/Akt (Protein Kinase B; PKB) pathway. This provides evidence that curcumin-triggered cellular antioxidant defenses can serve as an effective approach to anti-ageing intervention [73].

Moreover, it has been reported to increase the life span of fruit flies, mice and nematodes [74,75,76]. In fact, curcumin has been stated to improve and regulate the symptoms of age-related diseases such as atherosclerosis, cancer and diabetes [77,78]. Other than this, curcumin has been reported to show protective activity against chemotherapy-induced side effects and radiation-induced dermatitis in breast cancer patients [79,80]. Some studies have claimed that curcumin has anti-ageing potential because it can delay cellular senescence [81]. Cox et al. conducted a study to assess the effects of solid lipid curcumin on mood and cognition in healthy adults aged 60–85. In this study, subjects were examined for the effects of solid lipid curcumin formulation, i.e., 400 mg of Longvida^®^ for acute (1 and 3 h after a single dose), chronic (4 weeks) and acute-on-chronic (1 and 3 h after a single dose following regular treatment) dosing. The results obtained showed significant improvements in the working memory for both acute and chronic dosing. Additionally, it also decreased physical fatigue (measured per Chalder Fatigue Scale) as well as total and LDL cholesterol [82].

### 4.4. Emblica officinalis

*Emblica officinalis*, also known as Amla, is a member of the Phyllanthaceae family [83]. The churn of Amla is known for reducing cholesterol level and improving memory potential [84]. The consumption of Amla in the diet is effective in lowering the cholesterol level in the brain as well as in the body [85]. It has also been stated as a beneficial functional food for treating Alzheimer’s disease [86]. Draelos and colleagues conducted a double-blind study to evaluate the skin-lightening potential of a topical formulation comprising *E. officinalis* extract, glycolic acid and kojic acid. The study revealed that the topical formulation was 4% better than hydroquinone, due to which researchers claimed that the topical formulation could be an effective natural alternative for mild to moderate facial dyschromia [87]. Accumulation of free radicals in different tissues is associated with various stress-induced conditions leading to the progression of the process of ageing [88]. Tannoids obtained from *E. officinalis* also show a protective effect because of their antioxidant potential against the tardive dyskinesia rat model [89]. Moreover, the extract of *E. officinalis* shows antidepressant properties by inhibiting the activity of Gamma Amino Butyric Acid (GABA) and Monoamine oxidase-A (MAO-A) in consort with antioxidant activity in mice models [90].

### 4.5. Ginkgo biloba

*Ginkgo biloba*, also known as Gingko, is a functional food which improves the availability of oxygen in the tissues [91]. The leaves of Ginkgo have been reported to play a significant role in maintaining the blood flow and glucose level in the brain [92]. Moreover, it also improves the mental functioning of the brain [93]. Ascorbic acid, catechin, shikimic acid, lactone derivatives (ginkgolides) and isorhamnetin are some of the flavone glycosides, which are active scavengers of free radicals and are obtained from the extract of ginkgo leaves [94]. Huang conducted a study to assess the effect of *Gingko biloba* extract on the liver of the aged rat. The result revealed that administration of *Gingko biloba* extract reduced the level of liver metalloproteinase as well as malondialdehyde, and improved the SOD activity to minimize the oxidative stress [95]. Another study has revealed that the administration of *Ginkgo biloba* extract improves the cognitive function in aged female rats [96]. Even clinical studies have been conducted to assess the effect of *Gingko biloba* extract in the treatment of Alzheimer’s disease and cognitive function. Extensive analysis has revealed that the consumption of *Gingko biloba* extract improves the cognitive functioning of individuals who have mild dementia [97].

### 4.6. Glycyrrhiza glabra

*Glycyrrhiza glabra*, also known as licorice, is a member of the Fabaceae family [98]. The rhizomes, as well as roots of this plant, serve as a brain tonic which helps in regulating the blood sugar level [99]. Glycyrrhizin is the prime bioactive molecule obtained from this plant rich in antioxidants, which protects the brain from oxidative damage, maintains the normal functioning of the nervous system and improves the memory of the individual [100]. *Glycyrrhiza glabra* has a phenolic compound named “liquorice” which has antioxidant potential, due to which it is effective in the chelating of metal ions and the scavenging of free radicals [101]. It has been reported that *G. glabra* enhances the memory in the murine model of scopolamine-induced dementia [102]. Dhingra and colleagues also reported improvements in the memory of mice administered with *Glycyrrhiza glabra*. Three different doses, i.e., 75, 150 and 300 mg/kg p.o. of *Glycyrrhiza glabra* extracts, were administered for seven consecutive days. The result obtained showed that a dose of 150 mg/kg was effective in enhancing memory in the mice model [103].

### 4.7. Panax ginseng

*Panax ginseng*, also known as ginseng, is highly known for its medicinal value [104]. The bioactive molecule ginsenoside is obtained from the roots of this plant [105]. This bioactive molecule improves the resistance of the body against anxiety, fatigue, stress and trauma, and modulates the immune function [106]. Moreover, it also shows anti-stress properties and improves learning performance and memory [104]. A study reported an increase in the life span of juvenile mice with leukaemia upon the administration of ginseng [107]. Another study on *Panax ginseng* reported that it is able to decrease lipid peroxidation and improve antioxidant potential by reducing oxidative stress [108].

Moreover, double-blind clinical trials have confirmed that the consumption of ginseng improves the psychomotor performance of the individuals [109]. *Panax ginseng* has also been reported to have anti-melanogenic potential, and is associated with the activation of the foxo3a gene, also stated as the longevity gene [110]. Certain studies have reported that *Panax ginseng* prevents skin ageing. Furthermore, a randomized, placebo-controlled, double-blind study was conducted to assess the potential of both *Panax ginseng* and ginsenosides in preventing skin ageing. The result obtained from the study showed a significant reduction in wrinkle formation, and no participant showed an adverse reaction to the treatment [111].

## 5. Plant-Based Metabolites with Anti-Ageing and Medicinal Properties

### 5.1. Polyphenols

Plants are prime producers of secondary metabolites, especially polyphenolic compounds, and these are abundantly found in vegetables, fruits, cereals and beverages [112]. Polyphenols have intrigued researchers globally owing to their inherent properties, such as antioxidant potential, and their anticarcinogenic and anti-inflammatory action [113]. These characteristics enable polyphenolic compounds to be useful in the amelioration of various diseases, such as cancer, asthma, microbial infections, diabetes and cardiovascular diseases [114]. Studies have been conducted on numerous polyphenolic compounds, such as resveratrol, proanthocyanins and silymarin. They have been evaluated for their action on animal models subjected to DNA damage, oxidative stress and UV-induced skin irritation [115]. Moreover, these polyphenols, consolidated with sun protection cosmetic products, can effectively shield the skin from UV radiation-associated skin problems and aid in reducing the incidence of skin cancer [116]. Some polyphenols with therapeutic properties have been described below.

Resveratrol (Stilbenes) is a natural polyphenolic compound with antioxidant potential, and is present in the skin of peanuts and grapes [117]. In the last two decades, it has been a prime area of extensive research owing to its application as an anti-ageing ingredient [118]. Additionally, it exhibits anti-inflammatory action and radical scavenging properties, and can act as a chelating agent [119]. Studies have found it to be effective in the treatment of various diseases, including Alzheimer’s and cardiovascular disease [120]. Moreover, Bhat et al. stated that resveratrol possesses cancer chemo-preventive potential [121]. It also has a protective action against human skin, which was confirmed via the study conducted on HaCat cells exposed to nitric oxide free radical donor sodium nitroprusside [122]. Giardina and colleagues conducted an in vitro study on skin fibroblast to assess the efficacy of resveratrol on the proliferation and inhibition of collagen activity. The result obtained showed a dose-related increase in the proliferation rate of cells and substantial inhibition of collagenase activity [123]. Although it has been claimed that resveratrol has the potential to combat ageing at the cellular level and could be a breakthrough in anti-ageing and geriatric medicine, data supporting this claim in the human context are quite limited [124,125,126]. It has been well comprehended that resveratrol modulates mitochondrial biogenesis via stimulating Peroxisome proliferator-activated receptor gamma coactivator 1-alpha (PGC-1α), which further slows down the process of ageing and circumvents the chronic diseases [127,128].

Flavonoids (Phlorizin): Few plants have been found to synthesize phlorizin, a type of flavonoid [129]. It has been immensely exploited by pharmaceutical industries for more than a century, while also serving as a platform to evaluate physiological functioning [115]. Several studies have been conducted on the nutritional benefits of phlorizin. In a recent study, the anti-aging effects of phlorizin and phloretin were tested on murine senile osteoporosis models. The study revealed that phlorizin helped in the management of the ratio of receptor activator of nuclear factor kappa-Β ligand (RANKL) to osteoprotegerin (OPG), which is a biochemical marker of osteoporosis. Phlorizin also reduced the population of osteoclast cells expressing tartrate-resistant acid phosphatase (TRAP) [130]. Phlorizin is found at high concentrations in unripe apples. A preliminary study on human volunteers revealed the beneficial effects of unripe apples containing phlorizin in mitigating post-prandial hyperglycemia. The study was carried out on six healthy individuals and revealed that the consumption of unripe apples caused statistically significant reductions in post-prandial glucose response, as well as increased urinary glucose [131]. Mela and colleagues conducted a study to evaluate the effects of eight plant extracts as well as their combinations (apple (AE, 2.0 g), mulberry fruit (MFE, 1.5 g), elderberry (EE, 2.0 g), mulberry leaf (MLE, 1.0 g), turmeric (TE, 0.18 g), white bean (WBE, 3.0 g), EE  +  TE and AE  +  TE) on post-prandial insulin (PPI) and glucose (PPG) response. The results obtained from the study revealed that extracts of AE, MLE and MFE were effective in reducing PPI and PPG response [132]. Hyperglycemia has been reported to accelerate the aging process, which describes the potential of phlorizin in mitigating the effects of ageing, thereby improving the quality of life [133]. Many other plant extracts have emerged as potent sources of compounds with antioxidant potential [134]. Metabolites such as silymarin, genistein and apigenin have been found to impact the symptoms of skin ageing positively [91]. Still, no clinical or human trials have been conducted to unveil the real anti-ageing potential of phlorizin.

Apple Polyphenols: Apple is enriched with phytochemicals, especially polyphenols that exhibit immense antioxidant potential [135]. A wide range of polyphenolic compounds is found in apples, such as rutin, chlorogenic acid, catechin phloretin, epicatechin and proanthocyanidin B2 [136]. The daily consumption of apples has been portrayed to reduce the incidence of the occurrence of hypercholesterolemia and cardiovascular diseases [137]. Research studies have suggested that consuming apples can considerably lower the risk of lung cancer, especially in females [138]. Different studies have proven that apple is effective in impeding low-density lipoprotein (LDL) oxidation [137]. A study was conducted to evaluate the effects of apple polyphenols on the gene expression of CcO (cytochrome c oxidase) subunits III, CAT (catalase), Mth (methuselah), Rpn11, SOD and VIb. The result obtained from the study revealed that apple polyphenols increased the life span of fruit flies by 10%. Moreover, the downregulation of Mth, the upregulation of gene CAT, SOD1 and SOD2, and no significant change in the gene expression of CcO subunits, Rpn11 or VIb, were observed in the fruit flies [139]. Furthermore, concentrated apple juice has neuroprotective potential, confirmed via the studies conducted on normal aged mice and genetically compromised mice. Still, the anti-ageing potential of apple and its underlining mechanisms remain indefinable [51].

Blueberry Extract: Polyphenols are more abundantly found in blueberries than in other fruits and vegetables [140]. The high antioxidant potential of blueberry extracts has been associated with the amelioration of ageing symptoms [141]. Studies suggest that the regular consumption of blueberries can potentially enhance memory-related issues in elderly populations [142]. It has been stated that the consumption of blueberry extract slows down age-related functional and physiological deficits [143]. Galli and colleagues have found that supplementation with blueberry extract reversed the age-linked decline in the heat shock protein (HSP) of the hippocampal in rats [144]. Additionally, blueberries have been found to be effective in improving motor and cognitive behavior in aged rat models [145]. The life-prolonging potential of blueberry extracts has also been studied in fruit flies to understand the underlying mechanism. The results obtained from the study revealed that the incorporation of 5 mg/mL of blueberry extract into the diet significantly increased the lifespan of fruit flies by 10% [146].

Tea Catechins and Theaflavins: Tea has emerged as the most preferred beverage in the Asian subcontinent [147]. The beneficial aspects associated with the consumption of tea can be attributed to its inherent compounds, namely theaflavins and catechins [148]. Studies have shown the reduced oxidation of DNA molecules via regular intake of green or black tea [149]. Other in vivo studies on Drosophila have reported positive results concerning the increase in average life span by theaflavins and catechins [150]. Various published reports have stated that the consumption of oral tea polyphenols, as well as topical treatment with green tea, inhibits UV radiation- or chemical-induced skin tumorigenesis in various animal models [151]. Tea catechins and theaflavins possess both anti-inflammatory and anticarcinogenic properties [148]. Elmets and his team conducted a study to assess the effect of tea polyphenol extract on parameters linked with acute UV injury. For this, the skin of volunteers was first treated with green tea extract or its constituents, and treated sites were subjected to two minimal erythema doses of solar simulated radiation. Later, the skin was examined for the biochemical, clinical and histologic characteristics of UV-induced DNA damage. The results revealed that tea extract has a dose-dependent inhibitory effect on erythema response induced by UV irradiation. The histologic evaluation also showed a reduced number of Langerhans and sunburn cells [152].

Moreover, tea polyphenol extracts also reduced the DNA damage in the skin. Therefore, researchers stated that tea polyphenol extract could serve as a natural alternative for photoprotection [152]. Chiu and colleagues conducted a study to assess the effect of a combination therapy course of topical and oral green tea on the histological and clinical characteristics of photo-ageing. For this study, 40 women with rational photo-ageing were randomized either to a placebo regimen or a combination of 300 mg tea oral supplements (consumed twice daily) and 10% green tea cream for eight weeks. The results obtained from the study did not show any significant differences in the clinical characteristics of photo-ageing for the placebo or green tea-treated group. However, a histologic improvement in elastic tissue content was observed in the treated participants [153].

Black Rice Anthocyanins: Black rice is abundant in antioxidants, the supplementation of which has been proven to relieve symptoms in patients who have Alzheimer’s [10]. It also has an anticarcinogenic and anti-inflammatory effect [154]. It is also rich in anthocyanins, namely peonidin-3-glucoside and cyanidin-3-o glucoside [155]. Zuo and colleagues conducted a study of the potential of black rice in extending the lifespan of fruit flies. For determination, the effects on the gene expressions of CAT, Mth, Rpn11, SOD1 and SOD2 were evaluated. The result obtained from the study revealed that the consumption of 30 mg/dL of black rice anthocyanins prolonged the lifespan by 14% of the fruit flies. Moreover, the downregulated gene expression of Mth and the upregulated gene expression of CAT, Rpn11, SOD1 and SOD2 was recorded [156]. Huang et al. also conducted a study on a subacute ageing mice model to assess the effect of black rice anthocyanins, and found that black rice anthocyanins exhibit anti-ageing, anti-fatigue and anti-hypoxic properties [157].

### 5.2. Carotenoids

Carotenoids are vitamin A derivates, such as lycopene and β-carotene, which are known to possess high antioxidant potential as well as photoprotective characteristics [158]. β-carotene and lycopene can moderately improve skin texture [159].

β-Carotene is obtained from various plant sources, such as carrots, mangoes, papaya and pumpkins, among others [160]. It has emerged as a significant carotenoid owing to its characteristics, such as pro-vitamin-A activity, lipid radical scavenging activity and single oxygen quenching properties [161]. β-Carotene has been reported to avert erythema induced by UV rays and possess excellent photoprotection properties [162]. Reports have suggested the association of cellular ageing with low β-Carotene levels in plasma. A study conducted on 68 old-age subjects showed that β-carotene might modulate telomerase activity in older adults [163]. On the other hand, there are well-known ill effects of supplementary beta carotene for smokers, leading to the progression of lung cancer. A pioneering study in 1994 was published in the New England Journal of Medicine by the alpha tocopherol, beta carotene cancer prevention study group. This study reported that there was an unexpected observation of a greater incidence of lung cancer in men receiving supplementary beta-carotene, as opposed to those who did not [164].

Lycopene is a red carotene, carotenoid and phytochemical present in numerous fruits and vegetables such as papayas, watermelons, tomatoes, carrots and others [4]. It possesses a high single oxygen quenching potential, but lacks vitamin A activity [165]. Moreover, a study confirmed the role of lycopene in attenuating oxidative damage in tissues. Upon exposure to UV light, it was observed that more skin lycopene was destroyed in contrast to β-carotene [166]. Products of lycopene have also been reported to be effective against cancerous cells, in addition to their potential to significantly reduce MMP-1 activity, which is known to degrade collagen [167]. Both lycopene and β-carotene, dominant carotenoids found in human tissues and blood, are known to regulate skin properties [168]. In a very recently published paper, Cheng and co-workers reported that lycopene induces the base excision repair pathway in vitro in A549 cells. This study has opened a molecular pathway, which needs further investigation in vivo and in animal models [169].

### 5.3. Vitamins

Vitamin C is commonly known as ascorbic acid, and is a highly water-soluble vitamin [170]. This colorless compound has high antioxidant potential owing to its strong reducing nature [171]. The photosensitive ascorbic acid works best in a hydrophilic environment [172]. This crystalline compound is not synthesized in humans; therefore, it has to be taken in the regular diet [173]. Diets should be supplemented with vitamin C-rich sources, such as oranges, broccoli, brussels sprouts, green peppers, strawberries, kiwifruit and grapefruit, to avoid the vitamin C deficiency associated health problems like cardiovascular diseases, scurvy, and others [174]. Ascorbic acid has a high antioxidant potential and free radical-scavenging properties, which helps in preventing the oxidation of tissues, cell membranes and macromolecules (DNA and proteins) by free radicals [173].

Vitamin E is a fat-soluble membrane-bound compound which has high free radical-scavenging as well as antioxidant potential [175]. This nonenzymatic antioxidant is found in wheat germ oil, safflower oil, sunflower oil, vegetables, peanuts, corn, almonds, soy and meat [176]. A deficiency of vitamin E in the body may lead to the development of various health conditions in infants, such as dryness, papular erythema, depigmentation and oedema [177]. Vitamin E consumption helps in combating skin ageing symptoms due to its efficacy in preventing the peroxidation of lipids and the cross-connection of collagen fibers [4]. Vitamin E has been proven to relieve sunburn and UV-associated skin damage [178].

Both vitamins C and E work synergistically. For instance, when UV-induced molecules oxidize the cellular constituents, a chain reaction of lipid peroxidation starts in the membrane rich in polyunsaturated fatty acids. During this, d-α-tocopherol (antioxidant) gets oxidized to the tocopheroxyl radical and regenerates itself through ascorbic acid [179,180]. Different food sources such as corn, seeds, vegetable oils (sunflower oil and safflower oil) and soy are rich in tocopherol [4]. Moreover, the consumption of vitamin E from natural sources help against lipid peroxidation and collagen cross-linking, as both are associated with skin ageing. Additionally, topically applied vitamin E has also been reported to reduce chronic UVB-induced skin damage, erythema, sunburned cells and photocarcinogenesis [181,182]. A deficiency of vitamin E is also associated with a syndrome of edema with seborrheic changes, as well as depigmentation and dryness in premature infants [183]. Ekanayake-Mudiyanselage and Thiele, upon analyzing their study, stated that the level of vitamin E is dependent on the density of sebaceous glands in the skin. The oral supplementation of α-tocopherol for three weeks has been shown to cause a substantial increase in vitamin E levels in the sebaceous gland, especially on the face [184]. In a comparative study, the oral consumption of both vitamin C and E has been shown to improve the photoprotective effect in contrast to monotherapies [185]. Another study was conducted on 33 participants who received 100 or 180 mg vitamin C or placebo per day for four weeks. The result obtained from the study revealed that orally consumed vitamin C improved the radical scavenging activity of the skin by 22% (for 100 mg) and 37% (for 180 mg) from the baseline [87]. In the study by the alpha-tocopherol and beta carotene cancer prevention study group, it was found that vitamin E has insignificant effects on the prevention of lung cancer [164].

Nutraceuticals, functional foods and dietary supplements encompass a large group of compounds which are well known to improve health [186]. Functional foods have gained global attention owing to their impact on improving the symptoms of skin ageing [187]. Notably, fruits constitute an essential source of active metabolites used to curb skin ageing symptoms, as they are enriched with phenolic compounds, carotenoids and ascorbic acid, and possess high antioxidant potential [188]. The various plants and their components with anti-ageing potential are listed in Table 1.

## 6. Concluding Remarks

Ageing is a complex and progressive biological process, which gets affected by environmental and genetic factors. Nowadays, ageing is also linked with the consumption of an imbalanced diet deficient in many essential nutrients. Lately, nutraceuticals have gained appreciation and are being considered as a crucial element in improving life and providing antioxidant-containing molecules. Various vegetables and fruits contain antioxidant molecules with beneficial properties that can help in delaying the process of ageing. Moreover, these nutraceuticals do not show unwanted symptoms; instead, they have a beneficial impact on the digestive system. Therefore, nutraceuticals as food supplements have promising potential in combating as well as delaying the ageing process. The benefits associated with nutraceuticals prompts their incorporation into the diet for health benefits and long life. The current review meticulously summarizes the anti-ageing effects of plant-based supplements and plant-derived metabolites. Since most of the data have been obtained in vitro, caution is advised for inferring the clinical applicability of in vitro-tested molecules. Referencing, examining and confirming the human trial data is highly recommended.

## Figures and Tables

**Table 1:** Fruits and vegetable extracts and their phytochemicals with antiageing effects.

Common Name	Scientific Name	Study Conducted Region	Active Compounds	Biological Activities	Dose and Duration	Study Type	Experimental Models	References
Sweet orange	*Citrus sinensis* L.	Italy	Anthocyanins, flavanones, hydroxycinnamic acid and ascorbic acid	NF-B and AP-1 translocation and procaspase-3 cleavage	15 and 30 µg/mL for 7 h	In vitro	Human keratinocytes (HaCaT cell line)	[189]
Indian gooseberry	*Emblica officinalis* L.	Japan	Ascorbic acid, gallic Acid, elaeocarpusin	Inhibited type-I collagen collagenase, increase TIMP-1 level; Cellular proliferation inhibition and procollagen 1 protection against UVB-induced depletion by inhibition of UVB-induced MMP-1	(0–40 g/mL) for 48 h	In vitro	NB1RGB human skin fibroblasts	[190]
Indian gooseberry	*Emblica officinalis*	India	Ascorbic acid	Promotion of procollagen content and inhibition of matrix metalloproteinase levels in skin fibroblast	10–40 μg/mL for 24 h	In vitro	Fibroblast cell line (HS68 cell)	[191]
Cucumber	*Cucumis sativus* L.	India	Ascorbic acid	In vitro inhibition of hyaluronidase, elastase and MMP-1	20.98 and 6.14 μg/mL	In vitro assay	ND	[192]
Bitter gourd	*Momordica charantia* L.	China	Resveratrol	Anti-oxidative stress enhancement and UTH1, SKN7, SOD1 and SOD2 yeast gene expression regulation	1–3 μM for 12 h	In vitro	Yeast	[193]
Litchi, Rambutan, Tamarind	*Litchi chinensis*; *Nephelium lappaceum* L.; *Tamarindus indica*	Thailand	Ferulic acid, gallic acid, epigallocatechin	Suppression of melanin production in B16F10 melanoma cells through inhibition of tyrosinase and TRP-2; effectiveness for elastase and collagenase inhibition	0.05, 0.01 and 0.007 mg/mL for 72 h	In vitro	Human skin fibroblasts	[194]
Mandarin orange	*Citrus reticulata* Blanco	India	D-Limonene, n-Hexadecanoic acid	Collagenase and elastase inhibition, anti-enzymatic activity	NS	In vitro assay	ND	[195]
Snake fruit	*Salacca zalacca (Gaert.) Voss*	Indonesia	Chlorogenic acid	MMP-1 inhibition	NS	In silico	ND	[196]
Mandarin, Grapes	*Citrus sunki* Hort. ex Tanaka, *Citrus unshiu* Marcov, *Citrus sinensis* Osbeck, *Citrus reticulata* Blanco and *Vitis vinifera* L.	Republic of Korea	Narirutin, hesperidin, ascorbic acid	Increase in the expression levels of antioxidant enzymes; Reduction in skin thickness and wrinkle formation while elevating collagen level in an ultraviolet light B-exposed hairless mouse model	33, 100, 300 mg/kg for 10 weeks	In vitro and in vivo	Cell culture and mice	[197]
Carrot	*Daucus carota* L.	South Korea	Carrot glycoprotein	Neutralization of reactive oxygen, cell membrane protection	0.3, 0.5, 1 mg/mL	In vitro	Cell culture	[198]
Safflower Seed Oil	*Carthamus tinctorius*	France	Phenol	Inhibition in the collagenase assay, inhibition in the elastase assay	NS	In vitro assay	ND	[199]
Chinese quince	*Chaenomeles sinensis*	Japan	β-1,4-xyloglucan	Inhibition of the activity of dermal extracellular matrix proteases: Elastase and Collagenase	NS	In vitro assay	ND	[200]
Almonds	*Prunus dulcis*	California	α-tocopherol	Decreased wrinkle severity in postmenopausal females	340 kcal/day of almonds (58.9 g) for 16 weeks	Observational study	Human subjects	[201]
Maidenhair tree	*Ginkgo biloba* L.	China	kaempferol 3-O-β-D-glucopyranoside, isorhamnetin-3-O-glucoside, myricetin, ginkgolide A, bilobalide	Inhibition of ROS and MMP-1 degradation in human dermal fibroblasts	0.1, 0.2 mg/mL for 24 h	In vitro	Human dermal fibroblasts	[202]
Turmeric	*Curcuma longa*	India	Curcumin	Reduction in levels of C-reactive protein (CRP) an anti-ageing inflammatory marker	200 mg and 400 mg of Curcumin/kg bodyweight for six months	In vivo	Rat	[203]
Asian ginseng	*Panax ginseng*	Korea	Gingenoside	Promotion in collagen synthesis through the activation of transforming growth factor-β (TGF-β) in human skin fibroblast cells	0.05% PGLE for eight weeks	In vitro and In vivo	In vitro and human volunteer	[204]
Korean ginseng, mountain hawthorn	*Panax ginseng* Meyer and *Crataegus pinnatifida*	Republic of Korea	Ginsenoside	Protective effect against UVB-exposed photo-ageing of the skin by regulating procollagen type 1 and MMP-1 expression in NHDFs	100 μg/mL for 12 weeks	In vitro and Observational study	Human dermal fibroblasts, healthy human skin	[205]
Licorice	*Glycyrrhiza glabra* L.	Croatia	Glabridin and isoliquiritigenin	Tyrosinase and elastase inhibitory activity	NS	In vitro assay	ND	[101]
Siberian ginseng, touch-me-not	*Eleutherococcus senticosus*	Republic of Korea	Phlorizin	miR135b suppression improves the microenvironment and increases the proliferative potential of basal epidermal cells	NS	In vitro	Human keratinocytes	[206]
Marula	*Sclerocarya birrea*	South Africa	Quinic acid, catechin, epigallocatechin gallate and epicatechin gallate	Exhibited collagenase inhibition activities	100, 200 μg/mL	In vitro assay	ND	[207]
Lemon	*Citrus limon*	Japan	Eriocitrin (Polyphenols)	Increase in ageing-related scores (e.g., periophthalmic lesions) and delay in locomotor atrophy	4 mL and 6 mL/day/mouse	In vivo	Mice	[208]
Black rice	*Zizania aqatica*	China	Cyanidin -3-O-glucoside	Increases superoxide dismutase (SOD) and catalase (CAT), while decreases MDA and the activity of monoamine oxidase (MAO)	15, 30 and 60 mg/kg	In vivo	Mice	[209]
Green tea	*Camellia sinensis* L.	China	Epigallocatechin-3-gallate	Extension of lifespan through mitohormesis	50–300 μM for six days	In vivo	Caenorhabditis elegans	[210]
Orange Pekoe black tea	*Camellia sinensis* L.	Sri Lanka	Epigallocatechin gallate	Inhibition of elastase activity	NS	In vitro assay	ND	[211]
Banana	*Musa spaientum*	Korea	Corosolic acid	Inhibitory effects on MMPs activities	NS	In vitro assay	ND	[212]
Rice	*Oryza sativa*	Indonesia	Vanillin and coumaric acid	Elastase inhibitory activity	NS	In vitro assay	ND	[213]

NS: not specified; ND: not defined.
